# The Story of the Insane from Year to Year

**Published:** 1897-07-03

**Authors:** 


### THE STORY OF THE INSANE FROM YEAR TO YEAR.

Tenth Series.

### THE OXFORD COUNTY ASYLUM AT LITTLEMORE.

Here the numbers increased during the year from 535 to 541. There were 110 first admissions, 20 readmissions, 41 discharged recovered, 15 discharged relieved, and 59 died. The recoveries stand at 31 per cent. of the admissions and the deaths at 11 per cent. of the average number resident. Intemperance in drink caused the insanity in 9 of the year's admissions, and hereditary tendency in 27. Apoplexy is the assigned cause of death in 12 instances, and other brain diseases account for 22, consumption for 5, and pneumonia for 1. The asylum is full, like so many others, and to make room for the county patients it has been found necessary to discharge the Windsor patients. This will give space for 30 cases and breathing time for the committee, but, as Dr. Sankey remarks, the relief will only be temporary, and it is quite time that the visitors decided what they intend to do. Building additions is expensive work, but boarding out patients at other asylums is more expensive still, and it has many drawbacks besides the money one. Dr. Good, the assistant medical officer, delivers lectures to the attendants on nursing subjects, and 11 of his class have passed the examination of the Medico-Psychological Association. The weekly cost of maintenance is 7s. 6½d. per head. There is a balance of £191 in favour of the farm, but as usual we see no charge made for rent or patients' labour.

### EASTERN COUNTIES ASYLUM FOR IDIOTS, COLCHESTER.

This is not a rate-supported asylum, but depends for its existence on legacies and donations, and on the reception of a certain number of paying cases. There were 240 idiots in residence at the close of the year, being 6 more than on January 1st. There were 30 admissions, 10 discharges, and

14 deaths. The ages of the cases admitted range from 6 to 54 years. It is beyond doubt that if anything valuable is to be done in the education of idiots it should be commenced at an early age, whereas nineteen of the year's admissions were over 15 years of age, and five were over 20. Mr. Turner truly says that "it is much to be regretted that parents do not make an effort to secure the admission of their children at an earlier period of life." Although this education of idiot children is uphill work, much may be done, in many instances at least, to improve their lot, and nothing is more efficacious than developing the taste for various mechanical occupations. Mr. Turner says that the erection of suitable workshops is much needed, and we hope his idea will be carried out. Dr. Kirby, the medical officer, points out once again the frequency with which tuberculous disease attacks the idiot. Of fourteen deaths, consumption was the cause in 6, tubercular peritonitis in 2, and acute tuberculosis in 2.

### LAWN HOSPITAL FOR THE INSANE, LINCOLN.

The numbers in this hospital or asylum increased during the year from 72 to 81. There were 34 admissions, 9 discharged recovered, 4 discharged relieved, and 9 deaths. The percentage of recoveries on admissions was 25, and that of the deaths on the average number resident was 10. All the deaths were from ordinary causes. The asylum is evidently under careful management, and with numbers which never exceed 100 it is easy for all the arrangements to assume a sort of family character which would be difficult in larger institutions. Nearly 20 of the patients were sent to the seaside during the summer, and amusements of all kinds are liberally provided. There is a detached villa on the estate, which can be used for one or two patients who wish to have a pleasant and secluded residence. The hospital continues to do the work for which it came into existence, and many of the patients are received at low rates; but we think a table showing the various charges might be inserted in the report for next year. The committee say they cannot speak too highly of the excellent care and attention which Dr. Russell gives to the institution.

### HOLLOWAY SANATORIUM, VIRGINIA WATER.

On January 1st, 1896, this asylum contained 328 patients, and by the end of the year the number had risen to 351. There were 113 admissions, 24 readmissions, 58 recoveries, 26 discharged relieved, and 25 deaths. Excluding transfers the percentage of recoveries on admissions was 53·21, which is a high rate, and the deaths calculated on the average number resident were 7·28 per cent., all the deaths being from natural causes. Of the admissions, 41 owed their insanity to moral causes, such as mental anxiety, domestic trouble, and religion, 20 to intemperance in drink, and 25 to hereditary influence. One remarkable fact in connection with this sanatorium is the number of voluntary boarders resident. During 1896 there were 80 of these, making a total of 868 since the opening of the institution in 1885. We learn that no mechanical restraint has been used during the year and there was very little seclusion. Only one accident occurred—a fracture of the arm caused by accidental fall. Dr. Philipps laments, what others have done before him, that most forms of hydropathic treatment are being abandoned in asylums because they technically come under the heading of restraint; and he adds that had this mode of treatment been available the recovery rate would have been higher and suffering would have been avoided. Why such a soothing process should have to be described by the ugly word "restraint" is one of those mysteries which it is impossible to fathom as the treatment is precisely what any perfectly sane person would have to undergo in any hydropathic establishment. We are pleased to note that the charitable work of the hospital has not been forgotten. Not fewer than 129 patients pay 25s. a week or less; 16 pay nothing at all. Dr. Philipps contrasts the high number of nurses now considered necessary to look after a given number of patients and the small number considered sufficient 25 years ago, and he arrives at the conclusion that the huge nursing staff nowadays is not an unmixed advantage; and we are inclined to think there is something in what he says. Nothing is so conducive to mental recovery as constant occupation; the higher the number of nurses the less will be the probability of inducing patients to work. Dr. Philipps is a bold man. He actually admits a loss on his farming operations.

### THE BRISTOL ASYLUM.

Here, as almost everywhere else, we have to chronicle an increase in the numbers during 1896. There were 667 patients in residence on January 1st, and 734 on December 31st. The admissions were 187; readmissions, 25; recoveries, 58; discharged relieved, 11; and deaths, 76. The recoveries are 27·36 per cent. on the admissions, and the deaths are 10·81 per cent. on the average number resident. As causes of the insanity, domestic trouble, business anxieties, and such things, figure in six of the admissions only. Of the deaths 24 were from cerebral and spinal diseases, 11 from consumption, 6 from pneumonia, and 6 from bronchitis. In reading over our asylum reports we find frequent reference to the unfavourable nature of the cases admitted. It would seem that demented cases are much oftener sent to the county asylums than was the case thirty or even twenty years ago. Dr. Benham draws special attention to this, and he says that the "number of chronic dements continues to rise; some of them undoubtedly are well capable of treatment in the imbecile wards of a workhouse." This is quite true; but it is equally true that the unfortunate dement has had his last weeks or months greatly cheered by transference from the workhouse to the asylum. It is necessary to look on the reverse of a coin as well as the obverse. Systematic training of the nurses goes on in this asylum. Twenty have obtained the nursing certificate of the St. John's Society and thirteen that of the Medico-Psychological Association. The latter obtain a gratuity and a badge; and these, as Dr. Benham says, form inducements for all to compete. The farm is credited with a balance in its favour of £130, but we do not notice anything put down for rent of land, interest on capital, or patients' labour. The visitors express their appreciation of the services of the officers, and they pay a graceful tribute to Dr. Aveline, who lately left their service to take up the medical superintendency of the new Somerset Asylum. The table showing the average cost per head is not given, but the last published blue book gives the cost at 9s. 1⅝d.

### THE MONMOUTH ASYLUM, ABERGAVENNY.

There were 969 patients in residence on January 1st, 1896, and 975 on December 31st. The admissions were 160, readmissions 27, recoveries 62, relieved 10, and deaths 70. Put into percentages the recoveries stand at 33·1 on the admissions, and the deaths at 7·1 on the average number resident, both of these being below the average. Of the deaths 33 were from cerebral or spinal diseases, 8 to consumption, and 4 to pneumonia. Intemperance in drink was the assigned cause of insanity in 31 of the 187 admissions, and in 59 the insanity was either hereditary or congenital. The asylum is practically full, indeed, there are on the women's side of the house 13 more than there is proper accommodation for; but with the termination of the union between Monmouth, Brecon, and Radnor, there will be vacant accommodation for those patients chargeable to Monmouthshire. The committee express their high appreciation of the services of Dr. Glendenning and his able assistants. The average weekly cost of maintenance is for paupers a fraction over 7s. Some of the private patients seem to pay low sums, and others as high as 30s. a week.

